# Prediction of Endometrial Hyperplasia and Cancer among Premenopausal Women with Abnormal Uterine Bleeding

**DOI:** 10.1155/2019/8598152

**Published:** 2019-03-18

**Authors:** Luca Giannella, Lillo Bruno Cerami, Tiziano Setti, Ezio Bergamini, Fausto Boselli

**Affiliations:** ^1^Local Health Authority of Reggio Emilia-IRCCS, Obstetrics and Gynecology Unit, Cesare Magati Hospital, Scandiano, Italy; ^2^Mother-Infant Department, Institute of Obstetrics and Gynecology, University of Modena and Reggio Emilia, Modena, Italy

## Abstract

**Objective:**

To create a prediction model including clinical variables for the prediction of premalignant/malignant endometrial pathology in premenopausal women with abnormal uterine bleeding (AUB).

**Methods:**

This is an observational retrospective study including 240 premenopausal women with AUB referred to diagnostic hysteroscopy. Based on the presence of endometrial hyperplasia (EH) or cancer (EC), the women were divided into cases (EH/EC) and controls (no EH/EC). Univariate, stepwise logistic regression and ROC curve analysis were performed.

**Results:**

12 women had EH/EC (5%). Stepwise logistic regression analysis showed that EH/EC associated significantly with BMI ≥ 30 (OR=7.70, 95% CI 1.90 to 31.17), diabetes (OR=9.71, 95% CI 1.63 to 57.81), and a thickened endometrium (OR=1.20, 95% CI 1.08 to 1.34, criterion > 11 mm). The AUC was 0.854 (95% confidence intervals 0.803 to 0.896,* p*<0.0001). Considering the pretest probability for EH/EC of 5%, the prediction model with a positive likelihood ratio of 8.14 showed a posttest probability of 30%. The simultaneous presence of two or three risk factors was significantly more common in women with EH/EC than controls (50% vs. 6.6 and 25% vs. 0%, respectively,* p*<0.0001).

**Conclusion:**

When premenopausal vaginal bleeding occurs in diabetic obese women with ET > 11 mm, the percentage of premalignant/malignant endometrial pathology increases by 25%. It is likely that the simultaneous presence of several risk factors is necessary to significantly increase the probability of endometrial pathology.

## 1. Introduction

Abnormal uterine bleeding (AUB) is one of the most frequent reasons for a gynecological evaluation [1]. It can be caused by structural and nonstructural uterine disorders. According to FIGO system PALM-COEIN, the causes can be the following: polyp, adenomyosis, leiomyoma, malignancy, coagulopathy, ovulatory disfunction, endometrial, iatrogenic, or not yet classified [2]. Although in most cases AUB is not linked to a malignant or premalignant lesion, it should not be underestimated. We know that, in postmenopausal women with AUB, there is a risk of endometrial cancer of 10% [3, 4]. Then, with transvaginal ultrasonography showing an endometrial thickness (ET) < 4 mm, this risk falls below 1% [5].

In premenopausal women (PW) with AUB this risk stratification is not possible because the predictive performance of ET showed conflicting results [6–9]. In this group of women, other clinical variables are taken into account for the risk of EH/EC: obesity, nulliparity, age, infertility, intermenstrual bleeding, anovulation, and diabetes [10]. Based on the presence of these risk factors, some guidelines recommend endometrial biopsy as mandatory in women over the age of 40, or under the age of 40 in the presence of comorbidities [11, 12]. The UK NICE guidance recommends endometrial biopsy in PW with persistent intermenstrual bleeding (IB), or for women over the age of 45 with heavy menstrual bleeding (HMB), after the failure of medical treatment [13, 14].

Despite the presence of these guidelines, many studies in the literature have not given decisive results on the weight of the abovementioned risk factors [15–18]. Therefore, the correct management of PW with AUB is not entirely clear.

In a recent systematic literature review the risk of atypical endometrial hyperplasia (AEH) or cancer in PW with AUB was low: 1.31% [19]. These results raise the question about the appropriateness of endometrial biopsy in this population. In order to decrease false-positive cases, it is clear that endometrial biopsy should only be recommended in selected cases.

In this regard, in order to select a population most at risk of EH/EC, the objective of the present study was to create a predictive model including clinical variables for the prediction of premalignant/malignant endometrial pathology in PW with AUB.

## 2. Materials and Methods

This is an observational retrospective study including 240 premenopausal women with AUB referred to diagnostic hysteroscopy at nonuniversity (Scandiano) and university (Modena) hospital, Italy, from March 2010 to November 2014. As the present study was merely observational and included only analysis of data from routine measurements, with no additional or experimental interventions, institutional review board approval was not required. All patients provided written informed consent for the use of their data for research purposes prior to hysteroscopy.

AUB was defined by the presence of bleeding from the uterine corpus that was abnormal in volume, regularity, and/or timing, according to what was reported by women [2]. We excluded women with menopausal status (absence of menstruation for at least 12 months after the age of 40 years) [4]. When it was impossible to perform a hysteroscopy, that case was excluded from the study.

We included all those women who had a definitive histological diagnosis which we considered our reference standard. Vabra endometrial sampling for women without any intrauterine structural lesion; targeted biopsy in women with a suspected premalignant or malignant lesion; intrauterine lesion resection in women with polyps or myomas; all women with an atypical EH (AEH), as well as all women with an intrauterine malignancy, underwent a hysterectomy which represented our reference standard as definitive histological finding.

Based on the presence of endometrial hyperplasia (EH) or cancer (EC), the women were divided into cases (EH/EC) and controls (no EH/EC). Histological diagnosis of endometrial hyperplasia refers to the WHO 2014 classification: atypical and nonatypical [20].

All diagnostic hysteroscopies were performed without anesthesia in an outpatient setting and vaginoscopy with a saline solution as distension medium and using a 5 mm continuous-flow sheath with a viewing angle of 30°.

All data were collected from medical records. Patient characteristics taken into account were age (years), age at menarche (years), parity, body mass index (BMI = weight (kg)/height^2^ (m^2^)), presence of hypertension or diabetes, menstrual cycle phase, family history of breast and colorectal cancer, current hormonal therapy (progestogen only, combined oral contraceptives, and vaginal ring), smoking habit, endometrial thickness (mm), infertility, IB, tamoxifen users, and duration of AUB (expressed in months from its beginning).

The Kolmogorov-Smirnov test was used as the test for normal distribution. Continuous variables were expressed as median and interquartile range. Qualitative variables were expressed as numbers and percentages. Univariate logistic regression analysis was used to test all studied independent variables and the results were expressed as an odds ratio (OR) with 95% confidence intervals (CI). Multivariate logistic regression analysis was used to identify variables that associated significantly with EH/EC. For this, we included as explanatory variables all the variables that showed a* p* value ≤ 0.25 in the univariate model [21]. Multivariate analysis was performed using a stepwise method with an entrance and exit* p* value of 0.05/0.1. The predicted probabilities of the stepwise logistic regression analysis were then used to create a full ROC curve and to estimate the sensitivity, specificity, positive predictive value (PPV), negative predictive value (NPV), positive likelihood ratio (LR+), and negative likelihood ratio (LR-) of the prediction model. Receiver operating characteristic (ROC) curve analysis was also used to determine the optimal cut-off value of endometrial thickness for the prediction of EH/EC. After considering our disease prevalence (all cases of EH/EC) as the pretest probability for premalignant/malignant endometrial pathology, the likelihood ratio was used to calculate the posttest odds from the pretest odds of disease: posttest odds = pretest odds x likelihood ratio. The relation between odds and probability is as follows: odds = P/(1-P) and P = odds/(1+odds). Using these equations, we could calculate the posttest probability of disease from the pretest probability of disease [22]. Comparisons between categorical variables were performed by the chi-square test in order to assess if the presence of one or more risk factors associated with EH/EC.

All statistical analyses were performed using MedCalc Statistical Software version 18.10.2 (MedCalc Software bvba, Ostend, Belgium; http://www.medcalc.org; 2018). A *p* value of less than 0.05 was considered to be statistically significant.

## 3. Results

The data of 240 consecutive patients were analyzed retrospectively. Characteristics of the cohort are shown in Table 1.

Endometrial samples showed 3 women with EC (1.3%), 4 women with AEH (1.7%), 5 women with non-AEH (2%), 75 women with polyps (31.2%), 33 women with myomas (13.8%), and 120 women with negative results (50%). The prevalence of EH/EC was 5%.

Univariate logistic regression analysis showed that a BMI ≥ 30 (OR=8.13, 95% CI 2.34 to 28.21), the presence of diabetes (OR=12.33, 95% CI 2.64 to 57.4), or a thickened endometrium (expressed as a continuous variable in mm) (OR=1.15, 95% CI 1.05 to 1.26) associated with EH/EC (Table 2).

Multivariate analysis with the stepwise method showed that EH/EC associated significantly with BMI ≥ 30 (OR=7.70, 95% CI 1.90 to 31.17), diabetes (OR=9.71, 95% CI 1.63 to 57.81), and a thickened endometrium (OR=1.20, 95% CI 1.08 to 1.34) (Table 3). The variables that were not included in the model were menstruation cycle phase, menarche, nulliparity, tamoxifen users, and duration of AUB.

**Figure 1:**
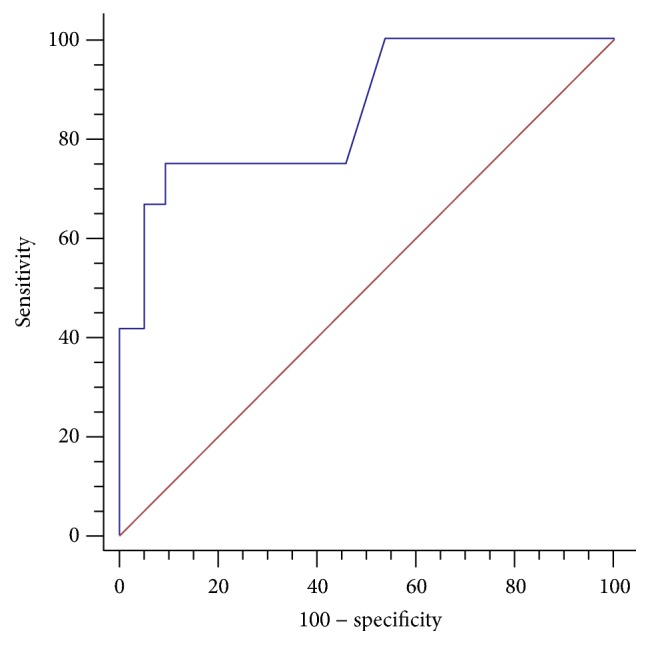
ROC curve associated with the prediction model. The area under the curve was 0.854 (95% CI 0.803 to 0.896,* p* < 0.0001).

The predicted probabilities of the logistic regression analysis were used to create a full ROC curve to estimate the sensitivity, specificity, PPV, NPV, LR+, and LR- of the prediction model. The AUC was 0.854 (95% confidence intervals 0.803 to 0.896,* p*<0.0001) (Figure 1). At the best cut-off value, sensitivity and specificity were 75.0% and 90.79%, respectively; the PPV and NPV were 30.0% and 98.6%, respectively; LR+ was 8.14 (with a pretest probability of 5% and posttest probability of 30.0%), and LR- was 0.28 (with a pretest probability of 5% and posttest probability of 1.5%).

Using ROC curve analysis, the best endometrial thickness cut-off value for the prediction of EH/EC was > 11 mm.

The simultaneous presence of two or three risk factors was significantly more common in women with EH/EC than controls (50% vs. 6.6 and 25% vs. 0%, respectively,* p*<0.0001) (Table 4).

## 4. Discussion

The present study showed that half of the PW with AUB have endocavitary uterine lesions. The prevalence of EC was 1.3%, while the prevalence of EH plus EC was 5%. These results are in line with previous data reported in the literature [19]. Despite the low percentage of EC, it should be emphasized that the risk of progression to cancer for nonatypical EH can reach up to 10% and up to 40% for AEH [23]. So, the risk of progression for premalignant endometrial pathology is not negligible. However, given the low disease prevalence, the risk of false positives or unnecessary examinations is high for this population. The present prediction model showed a moderate diagnostic accuracy for EH/EC with an AUC of 0.854. It included the presence of diabetes, BMI ≥ 30, and endometrial thickness > 11 mm with a posttest probability for EC of 30%, from a pretest probability of 5%. Furthermore, it would seem that the simultaneous presence of more than one risk factor significantly increases the percentage of EH/EC.

To date, the management of PW with AUB provides the need for endometrial sampling in women over 35–40 years of age, or under this cut-off value in the presence of comorbidities (anovulation, obesity, and diabetes) [1, 11, 12]. Another management option provides endometrial biopsy in women over 45 years of age with HMB, or in case of persistent IB [13, 14]. Although age seems to represent a crucial independent variable for the management of this population, many studies in the literature have not found this association [15–18]. In a very interesting paper, Wise et al. showed no association between age and EH/EC [24]. Likewise, Esmer et al. concluded that “the management of PW with AUB should be tailored to each patient regardless of age, incorporating all risk factors for malignant disease” [17]. Finally, Iram et al. in 2009 reported that their study, “the largest in the literature, suggests using the age of 45 years as a cut-off for sampling the endometrium in all PW with AUB. However, irregular menstrual bleeding justifies investigating women regardless of their age” [15]. With this conflicting evidence, the management of PW with AUB is a debated topic. Also our study, in line with the abovementioned data, showed no association between age and EH/EC.

Obesity and diabetes represent other risk factors for EH end EC in PW with AUB [1]. Many previous studies showed associations between these independent variables and endometrial pathology. Wise et al. showed that a BMI ≥ 30 was the most predictive factor for EH/EC with an adjusted odds ratio of 4.0 [24]. Guraslan et al. showed a strong association between obesity and AEH or EC [16]. Also the present study showed that diabetes and obesity are predictive factors for EH/EC with an AOR of 9.7 and 7.7, respectively.

The study of endometrial thickness as a predictive factor for endometrial pathology in PW with AUB is another debated topic with conflicting results in the literature. The biggest limitation of this investigation is given by the fact that the endometrium of premenopausal women already undergoes periodic changes of its thickness depending on the menstrual cycle phase. Therefore, its predictive performance is affected by this physiological occurrence. Several authors in their studies showed that endometrial thickness was of little value for the prediction of EH/EC [8, 9]. However, other studies showed that the presence of an ET > 8 mm in PW with AUB should provide an endometrial biopsy since the risk of endometrial pathology increases [6, 7]. Also Wise et al., when they included ET in the multivariate model, showed a strong association between EH/EC and ET > 11 mm (AOR=4.20, 95% CI 1.58–11.15) [24]. The same ET cut-off value (>11 mm) was associated with EH/EC in the present study.

Our prediction model including diabetes, ET > 11 mm, and BMI ≥ 30 showed a moderate diagnostic accuracy (AUC= 0.854) [25], with a LR+ of 8.14 and LR- of 0.28 at the best cut-off value. Considering our disease prevalence for EH/EC of 5%, the presence of the abovementioned variables increases the risk of endometrial pathology to 30%. Furthermore, when diabetes, ET > 11 mm, and BMI ≥ 30 are absent, the risk of EH/EC falls to 1.5%. This means that false positives and false negatives decrease. Far from saying that only women with these characteristics should perform an endometrial biopsy, the results of the present study seem to emphasize that women significantly at risk of EH/EC usually have the simultaneous presence of multiple risk factors and that, probably, different management based on the presence of only one risk factor does not significantly improve the diagnostic performance. Future studies with the objective of measuring this outcome will be able to assess the reliability of this hypothesis.

The present study has the great limitation of being retrospective. Given the low prevalence of EH/EC, a further weakness of the study was represented by the small number of subjects with premalignant or malignant endometrial pathology. A strength of the study is represented by the fact that each woman had a histological examination as a reference standard. Furthermore, all clinical variables included in the study were measurable in each woman (there were no missing data).

## 5. Conclusions

Limited to the study population, when premenopausal vaginal bleeding occurs in diabetic obese women with ET > 11 mm, the risk of premalignant/malignant endometrial pathology increases by 25%. It is likely that the simultaneous presence of several risk factors is necessary to significantly increase the probability of endometrial pathology.

## Figures and Tables

**Table 1:** Patient characteristics.

Variables	Study participants (n=240) n (%)
*Age (years)* Median (interquartile range)	44.0 (40.0 - 48.5)

*Menstrual cycle phase*	

Proliferative	135 (56.2)

Secretive	105 (43.8)

*Nulliparity*	63 (26.2)

*Menarche age (years)* Median (interquartile range)	12 (11.5 – 13.0)

*Current hormonal therapy *	38 (15.8)

*Body mass index*	

≥ 30	53 (22.1)

< 30	187 (77.9)

*Hypertension*	41 (17.1)

*Diabetes*	9 (3.7)

*Smoking habit*	71 (29.6)

*Endometrial thickness (mm)* Median (interquartile range)	10.0 (8.0 – 13.0)

*Infertility*	36 (15.0)

*Intermenstrual bleeding*	45 (18.8)

*Breast cancer family history*	9 (3.75)

*Colorectal cancer family history*	7 (2.9)

*Duration of AUB (months)* Median (interquartile range)	9 (8 – 23)

*Tamoxifen users*	2 (0.83)

*Histology*	

EC	3 (1.3)

AEH	4 (1.7)

Non-AEH	5 (2.0)

Polyp	75 (31.2)

Myoma	33 (13.8)

Negative	120 (50)

EC: endometrial cancer; AEH: atypical endometrial hyperplasia; AUB: abnormal uterine bleeding.

**Table 2:** Univariate logistic regression analysis showing associations with EH/EC.

Variables	OR	95% CI	*p value*
*Age (years)*	1.00	0.90 to 1.10	0.981

*Menstrual cycle phase*			
Secretive	0.41	0.10 to 1.56	0.191

*Nulliparity*			
Yes	3.00	0.93 to 9.67	0.065

*Menarche age (years)*	0.69	0.45 to 1.06	0.092

*Current hormonal therapy*			
Yes	1.83	0.47 to 7.12	0.378

*BMI*			
≥ 30	8.13	2.34 to 28.21	0.001

*Hypertension *			
Yes	0.97	0.20 to 4.59	0.968

*Diabetes*			
Yes	12.33	2.64 to 57.40	0.001

*Smoking habit*			
Yes	0.46	0.09 to 2.15	0.325

*Endometrial thickness (mm)*	1.15	1.05 to 1.26	0.002

*Infertility*			
Yes	1.96	0.50 to 7.65	0.327

*Intermenstrual bleeding*			
Yes	1.47	0.38 to 5.68	0.571

*Breast cancer family history*			
Yes	2.50	0.28 to 21.79	0.406

*Colorectal cancer family history*			
Yes	3.36	0.37 to 30.41	0.280

*Duration of AUB (months)*	0.92	0.84 to 1.01	0.105

*Tamoxifen users*			
Yes	6.81	0.65 to 70.97	0.108

EH/EC: endometrial hyperplasia/endometrial cancer; OR: odds ratio; CI: confidence intervals; BMI: body mass index; AUB: abnormal uterine bleeding.

**Table 3:** Multivariate logistic regression analysis for the prediction of EH/EC.

Variables	OR	95% CI	*p value* ^*∗*^
BMI ≥ 30	7.70	1.90 to 31.17	0.004

Diabetes = yes	9.71	1.63 to 57.81	0.012

Endometrial thickness criterion: > 11 mm	1.20	1.08 to 1.34	<0.001

*∗*Using stepwise method, variable not included in the model: nulliparity=yes, menarche, menstrual cycle phase=secretive, tamoxifen users=yes, and duration of AUB. EH/EC: endometrial hyperplasia/endometrial cancer; OR: odds ratio; CI: confidence intervals; BMI: body mass index.

**Table 4:** Presence of risk factors in women with and without EH/EC.

Risk factors (BMI>30; diabetes, ET>11 mm)	Women without EH/EC n (%)	Women with EH/EC n (%)	*p value*
			***<0.0001***

None	105 (46.1)	0 (0)	
1 risk factor	108 (47.3)	3 (25.0)	
2 risk factors	15 (6.6)	6 (50.0)	
3 risk factors	0 (0)	3 (25.0)	

EH/EC: endometrial hyperplasia/endometrial cancer; BMI: body mass index.

## Data Availability

The data used to support the findings of this study are available from the corresponding author upon request.
