# Corrigendum to “Prediction of Endometrial Hyperplasia and Cancer among Premenopausal Women with Abnormal Uterine Bleeding”

**DOI:** 10.1155/2020/3653414

**Published:** 2020-05-22

**Authors:** Luca Giannella, Lillo Bruno Cerami, Tiziano Setti, Ezio Bergamini, Fausto Boselli

**Affiliations:** ^1^Azienda USL-IRCCS di Reggio Emilia, Italy; ^2^Mother-Infant Department, Institute of Obstetrics and Gynecology, University of Modena and Reggio Emilia, Modena, Italy

In the article titled “Prediction of Endometrial Hyperplasia and Cancer among Premenopausal Women with Abnormal Uterine Bleeding” [1], authors Luca Giannella, Lillo Bruno Cerami, Tiziano Setti, and Ezio Bergamini were affiliated to the Local Health Authority of Reggio Emilia-IRCCS, Obstetrics and Gynecology Unit, Cesare Magati Hospital, Scandiano, Italy, which is incorrect. The correct affiliation for these authors is shown above.
